# Supplementary material for: Emerging variants develop total escape from potent monoclonal antibodies induced by BA.4/5 infection
Source: Nat Commun. 2024 Apr 16;15:3284. doi: 10.1038/s41467-024-47393-3 (PMC11021415; doi:10.1038/s41467-024-47393-3)
Supplement: Supplementary file 1 — Supplementary Information [file 41467_2024_47393_MOESM1_ESM.pdf]

# Supplementary Information

## Emerging variants develop total escape from potent monoclonal antibodies induced by BA.4/5 infection

**Authors**:

Chang Liu, Raksha Das, Aiste Dijokaite-Guraliuc, Daming Zhou, Alexander J. Mentzer, Piyada Supasa, Muneeswaran Selvaraj, Helen M.E. Duyvesteyn, Thomas G. Ritter, Nigel Temperton, Paul Klenerman, Susanna J. Dunachie, Neil G. Paterson, Mark A. Williams, David R. Hall, OPTIC consortium, ISARIC consortium, Elizabeth E. Fry, Juthathip Mongkolsapaya, Jingshan Ren, David I. Stuart, Gavin R Screaton

Contents:

Figures S1-S3 and Tables S1-S3

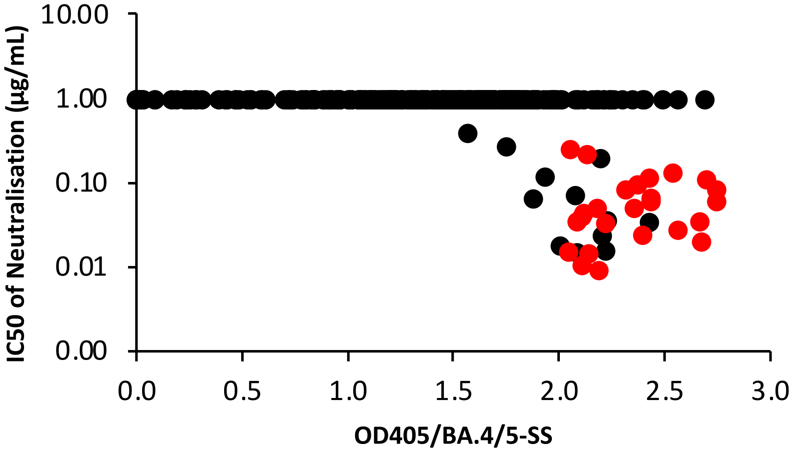


**Figure S1. IC50 of neutralisation against BA.5 and OD405 of binding on BA.4/5-SS.** Neutralisation of all antibodies isolated from BA.4/5 breakthrough samples were tested against BA.5 live virus, and their binding of BA.4/5 spike were assessed by ELISA. IC50 of neutralisation is plotted against OD405 illustrating their spike binding ability. 28 selected antibodies for further study were represented by red dots.

**a**

| | Victoria | BA.2 | BA.4 | BA.5 |
|---|---|---|---|---|
| **BA.4/5-1** | 14192 | 4942 | 3354 | 1859 |
| **BA.4/5-2** | 9519 | 2599 | 4117 | 1329 |
| **BA.4/5-3** | 112 | 107 | 381 | 659 |
| **BA.4/5-4** | 20087 | 5871 | 3774 | 3360 |
| **BA.4/5-5** | 17285 | 3560 | 3146 | 2356 |
| **BA.4/5-6** | 16860 | 6957 | 3686 | 4418 |
| **BA.4/5-7** | 7276 | 1214 | 2857 | 1385 |
| **BA.4/5-8** | 8111 | 1398 | 1327 | 949 |
| **BA.4/5-9** | 13618 | 5596 | 4442 | 3324 |
| **BA.4/5-10** | 17320 | 3481 | 4430 | 3176 |
| **BA.4/5-11** | 9742 | 1110 | 2154 | 1193 |

**b**

μg/mL

0.001 0.100 10

| | Victoria | Alpha | Beta | Gamma | Delta |
|---|---|---|---|---|---|
| **BA.4/5-1** | 0.004 | 0.008 | 0.006 | 0.008 | 0.017 |
| **BA.4/5-2** | 0.01 | 0.005 | 0.006 | 0.003 | 0.011 |
| **BA.4/5-3** | 0.019 | 0.01 | 0.008 | 0.007 | 0.003 |
| **BA.4/5-4** | 0.039 | 0.011 | 0.008 | 0.007 | 0.013 |
| **BA.4/5-6** | 0.008 | 0.011 | 0.009 | 0.004 | 0.013 |
| **BA.4/5-7** | 0.018 | 0.01 | 0.929 | 0.087 | 0.014 |
| **BA.4/5-8** | 0.015 | 0.008 | 0.003 | 0.004 | 0.013 |
| **BA.4/5-9** | 0.013 | 0.016 | 0.032 | 0.022 | 0.025 |
| **BA.4/5-10** | 0.011 | 0.011 | 0.005 | 0.002 | 0.005 |
| **BA.4/5-11** | 0.007 | 0.01 | 0.006 | 0.005 | 0.006 |
| **BA.4/5-12** | 0.029 | 0.023 | 0.014 | 0.013 | 0.017 |
| **BA.4/5-13** | 0.013 | 0.009 | 0.003 | 0.004 | 0.014 |
| **BA.4/5-15** | 0.017 | 0.008 | 0.014 | 0.012 | 0.019 |
| **BA.4/5-17** | 0.005 | 0.01 | 0.004 | 0.007 | 0.01 |
| **BA.4/5-18** | 0.009 | 0.008 | 0.007 | 0.012 | 0.008 |
| **BA.4/5-20** | 0.023 | 0.016 | 0.015 | 0.021 | 0.023 |
| **BA.4/5-21** | 0.002 | 0.006 | 0.004 | 0.004 | 0.009 |
| **BA.4/5-22** | 0.008 | 0.005 | 0.004 | 0.005 | 0.022 |
| **BA.4/5-23** | 0.032 | 0.014 | 0.012 | 0.006 | 0.01 |
| **BA.4/5-24** | 0.001 | 0.002 | 0.003 | 0.003 | 0.003 |
| **BA.4/5-25** | 0.021 | 0.01 | 0.003 | 0.002 | 0.004 |
| **BA.4/5-28** | 0.019 | 0.016 | 0.007 | 0.009 | 0.018 |
| **BA.4/5-31** | 0.013 | 0.011 | 0.008 | 0.015 | 0.013 |
| **BA.4/5-32** | 0.017 | 0.015 | 0.002 | 0.002 | 0.012 |
| **BA.4/5-33** | 10 | 10 | 10 | 0.076 | 10 |
| **BA.4/5-34** | 0.004 | 0.004 | 0.002 | 0.002 | 0.403 |
| **BA.4/5-35** | 0.009 | 0.007 | 0.003 | 0.002 | 0.01 |
| **BA.4/5-36** | 10 | 0.019 | 10 | 10 | 10 |

**Figure S2**. **FRNT50 of BA.4/5 breakthrough serum samples and heatmaps of antibody IC50 neutralisation titres**. **a** FRNT50 titres of BA.4/5 breakthrough infection serum samples against Victoria, BA.2, BA.4, and BA.5 pseudovirus.
**b** Heatmap of IC50s of potent BA.4/5 mAbs against pseudoviruses expressing early variants of concern S sequences.

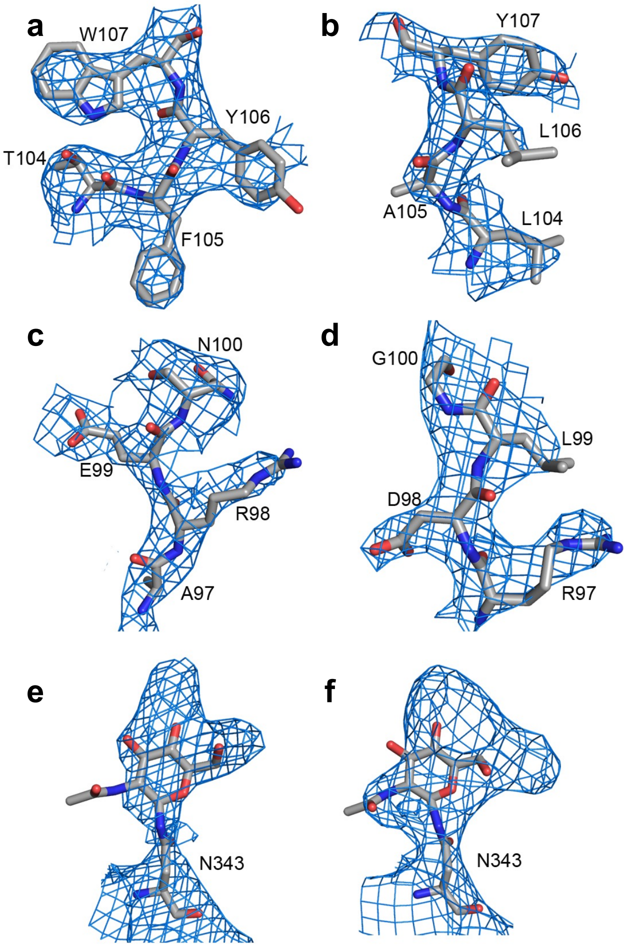


**Figure S3. Electron density maps. a-d** Electron density maps for a portion of CDR-H3 of BA.4/5-1, BA.4/5-2, BA.4/5-9 and BA.4/5-35 respectively. The maps are contoured at 1.5 σ except BA.4/5-2 which is contoured at 1.0 σ. **e**, **f** Electron density contoured at 1.0 σ for the N-linked oligosaccharide at N343 of the RBD in RBD/BA.4/5-1 and RBD/BA.4/5-9 respectively.

| Ab id. | Protein-Specific | Heavy Chain: V-REGION Nb of AA changes | Heavy Chain: V-GENE and allele | Heavy Chain: J-GENE and allele | Heavy Chain: D-GENE and allele | Light Chain | Light Chain: V-REGION Nb of AA changes | Light Chain: V-GENE and allele | Light Chain: J-GENE and allele |
|---|---|---|---|---|---|---|---|---|---|
| BA.4/5-1 | RBD | 5 | 4-39*01 F | 4*02 F | 1-26*01 F | K | 7 | 1-NL1*01 F | 2*01 F |
| BA.4/5-2 | RBD | 14 | 3-30*03 F, or 3-30*18 F or 3-30-5*01 F | 6*02 F | 2-15*01 F | K | 5 | 3-11*01 F | 3*01 F |
| BA.4/5-3 | RBD | 16 | 4-61*02 F, or 4-61*11 | 4*02 F | 1-26*01 F | K | 9 | 1-5*01 F | 1*01 F |
| BA.4/5-4 | RBD | 11 | 1-3*01 F, or 1-3*04 | 6*03 F | 6-25*01 F | K | 5 | 1-5*01 F | 4*01 F |
| BA.4/5-6 | RBD | 10 | 3-53*04 F | 4*02 F | 4-17*01 F | K | 9 | 3-20*01 F | 2*02 |
| BA.4/5-7 | RBD | 14 | 3-53*02 F | 4*02 F | 4-17*01 F | K | 5 | 3-15*01 F | 1*01 F |
| BA.4/5-8 | RBD | 6 | 3-66*02 F | 6*02 F | 1-26*01 F | K | 4 | 1-33*01 F, or 1D-33*01 F | 2*01 F |
| BA.4/5-9 | RBD | 13 | 1-46*01 F, or 1-46*02 F or 1-46*03 F | 4*02 F | 4-23*01 ORF | K | 6 | 3-11*01 F | 2*03 |
| BA.4/5-10 | RBD | 2 | 3-66*02 F | 6*02 F | 1-26*01 F | K | 5 | 1-33*01 F, or 1D-33*01 F | 2*01 F |
| BA.4/5-11 | RBD | 6 | 3-33*01 F, or 3-33*03 F or 3-33*06 F | 3*02 F | 3-22*01 F | K | 3 | 1-39*01 F, or 1D-39*01 F | 2*01 F |
| BA.4/5-12 | RBD | 9 | 3-30*03 F, or, 3-30*18 F, or 3-30-5*01 F | 6*02 F | 3-9*01 F | K | 8 | 1-33*01 F,or 1D-33*01 F | 5*01 F |
| BA.4/5-13 | RBD | 9 | 1-69*01 F, or 1-69D*01 F | 6*02 F | 5-18*01 F | λ | 14 | 1-33*01 F, or 1D-33*01 F | 3*01 F |
| BA.4/5-15 | RBD | 6 | 3-9*01 F | 6*02 F | 3-9*01 F | λ | 9 | 1-44*01 F | 1*01 F |
| BA.4/5-17 | RBD | 8 | 3-53*04 F | 6*02 F | 6-19*01 F | λ | 4 | 1-51*01 F | 1*01 F |
| BA.4/5-18 | RBD | 11 | 1-69*01 F, or 1-69D*01 F | 6*02 F | 5-18*01 F | K | 18 | 1-33*01 F, or 1D-33*01 F | 3*01 F |
| BA.4/5-20 | RBD | 11 | 3-9*01 F | 6*02 F | 3-9*01 F | λ | 8 | 1-44*01 F | 1*01 F |
| BA.4/5-21 | RBD | 11 | 3-9*01 F | 4*02 F | 3-22*01 F | K | 12 | 1-9*01 F | 4*01 F |
| BA.4/5-22 | RBD | 13 | 1-69*01 F, or 1-69D*01 F | 4*02 F | 5-12*01 F | K | 11 | 2-30*01 F | 1*01 F |
| BA.4/5-23 | RBD | 15 | 3-53*01 F | 4*02 F | 4-17*01 F | K | 12 | 3-20*01 F | 1*01 F |
| BA.4/5-24 | RBD | 9 | 3-9*01 F | 5*02 F | 3-22*01 F | K | 17 | 1-9*01 F | 3*01 F, or 4*01 F |
| BA.4/5-25 | RBD | 16 | 1-69*09 F | 4*02 F | 5-18*01 F | K | 9 | 1-5*01 F | 2*02 |
| BA.4/5-28 | RBD | 10 | 3-66*01 F, or 3-66*04 F | 6*02 F | 1-1*01 F | λ | 16 | 3-21*04 F | 2*01 F, or 3*01 F or 3*02 F |
| BA.4/5-31 | RBD | 6 | 4-39*01 F | 4*02 F | 1-26*01 F | K | 15 | 1-NL1*01 F | 2*01 F |
| BA.4/5-32 | RBD | 12 | 3-66*02 F | 3*01 F | 3-10*02 F | λ | 10 | 3-21*02 F | 1*01 F |
| BA.4/5-33 | NTD | 13 | 3-23*04 F | 4*02 F | 5-18*01 F | K | 5 | 3-15*01 F | 2*01 F |
| BA.4/5-34 | RBD | 12 | 1-69*01 F, or1-69D*01 F | 4*02 F | 5-12*01 F | K | 8 | 2-30*01 F | 1*01 F |
| BA.4/5-35 | RBD | 12 | 3-66*01 F, or 3-66*04 F | 4*02 F | 1-26*01 F | K | 6 | 3-15*01 F | 2*01 F |
| BA.4/5-36 | NTD | 13 | 4-4*02 F | 3*02 F | 2-21*01 F | K | 16 | 1-5*01 F | 1*01 F |

**Table S1.** Ig variable gene usage for BA.4/5 mAb

| **Structure** | Delta-RBD/BA.4/5-1/EY6A | Delta-RBD/BA.4/5-2/Beta-49 | Delta-RBD/BA.4/5-9 | Delta-RBD/BA.4/5-35 |
|---|---|---|---|---|
| **PDB ID** | 8CBD | 8CBE | 8QZR | 8CMA |
| **Data collection** | | | | |
| Space group | *C*2 | $P2_12_12$ | *I*23 | *C*2 |
| *a*, *b*, *c* (Å) | 242.0, 139.4, 168.5 | 179.9, 147.0, 52.2 | 251.6, 251.6, 251.6 | 195.8, 85.1, 57.0 |
| α, β, γ (°) | 90, 115.6, 90 | 90, 90, 90 | 90, 90, 90 | 90, 102.0, 90 |
| Resolution (Å) | 75–3.52 (3.58–3.52)[a] | 60–3.16 (3.21–3.16) | 59-3.77 (3.84–3.77) | 48–3.29 (3.35–3.29) |
| $R_{merge}$ | 0.325 (---) | 0.345 (---) | 0.414 (---) | 0.298 (---) |
| $R_{pim}$ | 0.133 (1.192) | 0.098 (0.994) | 0.065 (1.027) | 0.121 (1.270) |
| $I/\sigma(I)$ | 4.9 (0.4) | 6.1 (0.5) | 8.0 (0.4) | 5.6 (0.5) |
| $CC_{1/2}$ | 0.984 (0.351) | 0.991 (0.322) | 0.998 (0.351) | 0.982 (0.340) |
| Completeness (%) | 99.7 (98.4) | 100 (99.2) | 100 (100) | 98.9 (94.6) |
| Redundancy | 7.1 (6.7) | 13.3 (13.2) | 41.8 (42.8) | 7.0 (6.8) |
| **Refinement** | | | | |
| Resolution (Å) | 75–3.52 | 57–3.16 | 59–3.77 | 48–3.29 |
| No. reflections | 59185/3023 | 23351/1206 | 25675/1319 | 13161/681 |
| $R_{work}$ / $R_{free}$ | 0.219/0.267 | 0.227/0.268 | 0.251/0.30 | 0.231/0.268 |
| No. atoms | | | | |
| Protein | 24090 | 8087 | 8671 | 4718 |
| Ligand/ion/water | 14 | 49 | 38 | 14 |
| *B* factors ($Å^2$) | | | | |
| Protein | 169 | 108 | 196 | 125 |
| Ligand/ion/water | 207 | 123 | 230 | 192 |
| r.m.s. deviations | | | | |
| Bond lengths (Å) | 0.003 | 0.002 | 0.002 | 0.002 |
| Bond angles (°) | 0.5 | 0.5 | 0.5 | 0.4 |

[a] Values in parentheses are for highest-resolution shell.

**Table S2.** Data collection, structure determination and refinement statistics

| | sex | Age range | 1st Vaccine Date (DD/MM/YY) | Vaccine 1 | 2nd Vaccine Date (DD/MM/YY) | Vaccine2 | 3rd Vaccine Date (DD/MM/YY) | Vaccine3 | Date of Positive Test (DD/MM/YY) | Variant Infected | Days after infection |
|---|---|---|---|---|---|---|---|---|---|---|---|
| **BA.4/5-1** | M | 30-39 | 09/08/2021 | Pfizer | Pfizer | 31/12/2021 | MODERNA | NA | 07/06/2022 | BA.4 | 30 |
| **BA.4/5-2** | F | 50-59 | 01/09/2021 | Pfizer | 25/03/2021 | Pfizer | 11/11/2021 | Pfizer | 14/06/2022 | BA.5 | 24 |
| **BA.4/5-3** | F | 90-99 | NA | NA | NA | NA | NA | NA | 26/06/2022 | BA.5 | 30 |
| **BA.4/5-4** | M | 60-69 | 03/09/2021 | AZ | 29/05/2021 | AZ | 30/11/2021 | Moderna | 22/06/2022 | BA.5 | 35 |
| **BA.4/5-5** | F | 40-49 | 20/05/2021 | Pfizer | 31/07/2021 | Pfizer | 22/12/2022 | Moderna | 24/06/2022 | BA.4 | 36 |
| **BA.4/5-6** | M | 40-49 | 17/05/2021 | Pfizer | 12/07/2021 | Pfizer | NA | NA | 11/06/2022 | BA.5 | 49 |
| **BA.4/5-7** | M | 30-39 | 04/06/2021 | Pfizer | 31/07/2021 | Pfizer | NA | NA | 25/06/2022 | BA.5 | 36 |
| **BA.4/5-8** | F | 20-29 | 15/01/2021 | Pfizer | 18/03/2022 | Pfizer | 18/11/2021 | Pfizer | 22/06/2022 | BA.5 | 41 |
| **BA.4/5-9** | F | 50-59 | 12/12/2020 | Pfizer | 01/02/2021 | Pfizer | 02/10/2021 | Pfizer | 22/06/2022 | BA.5 | 43 |
| **BA.4/5-10** | F | 20-29 | 27/06/2021 | Pfizer | 30/08/2021 | Pfizer | 24/12/2021 | Pfizer | 26/06/2022 | BA.4 | 41 |
| **BA.4/5-11** | M | 40-49 | 26/02/2021 | AZ | 06/07/2021 | AZ | 06/12/2021 | Moderna | 23/06/2022 | BA.5 | 45 |

**Table S3.** Information of BA.4/5 infected patient samples
