# Supplementary material for: Emerging variants develop total escape from potent monoclonal antibodies induced by BA.4/5 infection
Source: Nat Commun. 2024 Apr 16;15:3284. doi: 10.1038/s41467-024-47393-3 (PMC11021415; doi:10.1038/s41467-024-47393-3)
Supplement: Supplementary file 3 — Description of Additional Supplementary Files [file 41467_2024_47393_MOESM3_ESM.pdf]

Description of Additional Supplementary Files

File Name: Supplementary Data 1
Description: Primers
