# Supplementary material for: Emerging variants develop total escape from potent monoclonal antibodies induced by BA.4/5 infection
Source: Nat Commun. 2024 Apr 16;15:3284. doi: 10.1038/s41467-024-47393-3 (PMC11021415; doi:10.1038/s41467-024-47393-3)
Supplement: Supplementary file 5 — Reporting Summary [file 41467_2024_47393_MOESM5_ESM.pdf]

nature portfolio

Corresponding author(s): David Stuart, Gavin Screaton

Last updated by author(s): Dec 5, 2023

Reporting Summary

Nature Portfolio wishes to improve the reproducibility of the work that we publish. This form provides structure for consistency and transparency in reporting. For further information on Nature Portfolio policies, see our Editorial Policies and the Editorial Policy Checklist.

Statistics

For all statistical analyses, confirm that the following items are present in the figure legend, table legend, main text, or Methods section.

n/a	Confirmed	
☐	☒	The exact sample size (*n*) for each experimental group/condition, given as a discrete number and unit of measurement
☐	☒	A statement on whether measurements were taken from distinct samples or whether the same sample was measured repeatedly
☐	☒	The statistical test(s) used AND whether they are one- or two-sided<br>*Only common tests should be described solely by name; describe more complex techniques in the Methods section.*
☒	☐	A description of all covariates tested
☒	☐	A description of any assumptions or corrections, such as tests of normality and adjustment for multiple comparisons
☐	☒	A full description of the statistical parameters including central tendency (e.g. means) or other basic estimates (e.g. regression coefficient) AND variation (e.g. standard deviation) or associated estimates of uncertainty (e.g. confidence intervals)
☐	☒	For null hypothesis testing, the test statistic (e.g. *F*, *t*, *r*) with confidence intervals, effect sizes, degrees of freedom and *P* value noted<br>*Give P values as exact values whenever suitable.*
☒	☐	For Bayesian analysis, information on the choice of priors and Markov chain Monte Carlo settings
☒	☐	For hierarchical and complex designs, identification of the appropriate level for tests and full reporting of outcomes
☒	☐	Estimates of effect sizes (e.g. Cohen's *d*, Pearson's *r*), indicating how they were calculated

Our web collection on statistics for biologists contains articles on many of the points above.

Software and code

Policy information about availability of computer code

Data collection	UDC at Diamond. Xia2-dials
Data analysis	Xia2-Dials, Phaser, Coot, Phenix

For manuscripts utilizing custom algorithms or software that are central to the research but not yet described in published literature, software must be made available to editors and reviewers. We strongly encourage code deposition in a community repository (e.g. GitHub). See the Nature Portfolio guidelines for submitting code & software for further information.

Data

Policy information about availability of data

All manuscripts must include a data availability statement. This statement should provide the following information, where applicable:

- Accession codes, unique identifiers, or web links for publicly available datasets
- A description of any restrictions on data availability
- For clinical datasets or third party data, please ensure that the statement adheres to our policy

The structures are available form the PDB with accession codes: 8CBD, 8CBE, 8QZR, 8CMA.

Research involving human participants, their data, or biological material

Policy information about studies with human participants or human data. See also policy information about sex, gender (identity/presentation), and sexual orientation and race, ethnicity and racism.

Reporting on sex and gender	Individuals participating in the study are referenced to by sex only. Individuals were not approached, consented, or used in the study based on sex. Consent was gained to collect data on sex with no specification for use of individual level data relating to sex, but data could be shared as long as remained psudonymised.
Reporting on race, ethnicity, or other socially relevant groupings	No reference to ethnicity is made in relation to the participants included in this study.
Population characteristics	N/A
Recruitment	Individuals participating in studies such as the "ISARIC/WHO Clinical Characterisation Protocol for Severe Emerging Infections" [Oxford REC C, reference 13/SC/0149], or the "Gastro-intestinal illness in Oxford: COVID sub study" [Sheffield REC, reference: 16/YH/0247]with confirmed BA.4 or BA.5 infection were included in this study. Alternatively, individuals managed in Oxford University NHS Foundation Trust with confirmed BA.4 or BA.5 infection were approached and asked whether they would be interested in participating through the "Innate and adaptive immunity against SARS-CoV-2 in healthcare worker family and household members" protocol (approved by the University of Oxford Central University Research Ethics Committee). If consenting, the were sampled and included in this study.
Ethics oversight	The committees approving the individual studied are detailed in the 'Recruitment' section above.

Note that full information on the approval of the study protocol must also be provided in the manuscript.

Field-specific reporting

Please select the one below that is the best fit for your research. If you are not sure, read the appropriate sections before making your selection.

☒ Life sciences ☐ Behavioural & social sciences ☐ Ecological, evolutionary & environmental sciences

For a reference copy of the document with all sections, see nature.com/documents/nr-reporting-summary-flat.pdf

Life sciences study design

All studies must disclose on these points even when the disclosure is negative.

Sample size	N/A
Data exclusions	No exclusions
Replication	Structural results are not replicated but the methods are subjected to rigorous validation.
Randomization	N/A
Blinding	N/A

Reporting for specific materials, systems and methods

We require information from authors about some types of materials, experimental systems and methods used in many studies. Here, indicate whether each material, system or method listed is relevant to your study. If you are not sure if a list item applies to your research, read the appropriate section before selecting a response.

Materials & experimental systems

n/a	Involved in the study	
☐	☒	Antibodies
☐	☒	Eukaryotic cell lines
☒	☐	Palaeontology and archaeology
☒	☐	Animals and other organisms
☒	☐	Clinical data
☒	☐	Dual use research of concern
☒	☐	Plants

Methods

n/a	Involved in the study	
☒	☐	ChIP-seq
☐	☒	Flow cytometry
☒	☐	MRI-based neuroimaging

Antibodies

Antibodies used	Antibodies were produced ourselves, as described.
Validation	As described.

Eukaryotic cell lines

Policy information about cell lines and Sex and Gender in Research

Cell line source(s)	HEK293 cells, source ATCC, Cat#CRL-3216; HEK293T/17 cells, source ATCC, Cat#CRL-11268™; Expi293F, source Gibco, Cat#A14527; Vero cells, source ATCC, Cat#CCL81; VeroE6/TMPRSS2, source NIBSC, Cat#100978
Authentication	Not authenticated
Mycoplasma contamination	Not tested
Commonly misidentified lines (See ICLAC register)	No commonly misidentified cell lines in this study.

Plants

Seed stocks	*Report on the source of all seed stocks or other plant material used. If applicable, state the seed stock centre and catalogue number. If plant specimens were collected from the field, describe the collection location, date and sampling procedures.*
Novel plant genotypes	*Describe the methods by which all novel plant genotypes were produced. This includes those generated by transgenic approaches, gene editing, chemical/radiation-based mutagenesis and hybridization. For transgenic lines, describe the transformation method, the number of independent lines analyzed and the generation upon which experiments were performed. For gene-edited lines, describe the editor used, the endogenous sequence targeted for editing, the targeting guide RNA sequence (if applicable) and how the editor was applied.*
Authentication	*Describe any authentication procedures for each seed stock used or novel genotype generated. Describe any experiments used to assess the effect of a mutation and, where applicable, how potential secondary effects (e.g. second site T-DNA insertions, mosiacism, off-target gene editing) were examined.*

Flow Cytometry

Plots

Confirm that:

- [x] The axis labels state the marker and fluorochrome used (e.g. CD4-FITC).
- [x] The axis scales are clearly visible. Include numbers along axes only for bottom left plot of group (a 'group' is an analysis of identical markers).
- [x] All plots are contour plots with outliers or pseudocolor plots.
- [ ] A numerical value for number of cells or percentage (with statistics) is provided.

Methodology

Sample preparation	PBMC were isolated from PCR-confirmed, BA.4/5 infected convalescent blood samples. PBMC were stained with LIVE/DEAD Fixable Aqua dye (Invitrogen) followed by recombinant trimeric S-twin-Strep of BA.4/5. Cells were then incubated with CD3-FITC, CD14-FITC, CD16-FITC, CD56-FITC, IgM-FITC, IgA-FITC, IgD-FITC, IgG-BV786 and CD19-BUV395, along with Strep-MAB-DY549 to stain the twin strep tag of the S protein.
Instrument	BD FACSAria Fusion sorter
Software	DIVA
Cell population abundance	100% sorted cells were relevant cell population. As single cell sorting was used in this study, the purify of the samples was not tested by FACS, but PCR process following sorting confirmed the purity of the population.
Gating strategy	The preliminary FSC/SSC gates of the staining population was identified by the general location of lymphocytes as shown in literatures; IgG+ memory B cells were gated as CD19+, IgG+, CD3-, CD14-, CD56-, CD16-, IgM-, IgA- and IgD-, and S+ was further selected; the boundaries between 'positive' and 'negative' staining cell populations were defined by negative control samples.

- [x] Tick this box to confirm that a figure exemplifying the gating strategy is provided in the Supplementary Information.
